# Dual-stress-responsive-pathways prevent HCC initiation via p53 suppression of MTHFD1L-RNA m^6^A-elicited autonomous growth and immune evasion

**DOI:** 10.21203/rs.3.rs-9474831/v1

**Published:** 2026-04-30

**Authors:** Hua Lu, Yi-Wei Zhang, Gang peng, Wenjuan Liao, Ji Hoon Jung, Caiyue Li, Yunlong Liu, Hong Gao, Yitian Zha, Nathan Pham, Stephen Meaderis, Evan Eslter, Yanping Zhang, Debanjan Dhar, Shelya Zeng

**Affiliations:** Tulane University School of Medicine; Tulane University School of Medicine; Indiana University School of Medicine; Tulane University School of Medicine; Tulane University School of Medicine; Tulane University School of Medicine; IUPUI; Indiana University School of Medicine; Tulane University School of Medicine; Tulane University School of Medicine; Tulane University School of Medicine; Tulane University School of Medicine; UNC; University of California at San Diego; Tulane University- school of medicine

**Keywords:** p53, MDM2, MDMX, Mthfdl, HCC, NASH, cirrhosis, one-carbon metabolism (1CM), m6A, Snail, B2m, antigen presentation, immune evasion, HCC initiating cell (HCICs), liver cancer initiation

## Abstract

Hepatocellular carcinoma (HCC) originates from damaged hepatocytes in chronic liver injury. Though HCC development requires oncoproteins-mediated p53 inhibition, both genetic deletion and continous activation of p53 specifically in mouse hepatocytes are shown to promote liver tumorigenesis, suggesting that chronic liver injury firstly activates p53, and p53 activity needs to be dynamically regulated for preventing hepatocarcinogenesis. Yet, it remains unaddressed how liver injury stresses trigger signaling pathways to rapidly activate p53 prior to its inactivation in a physiological setting. Two pathways, ribosomal proteins (RPs)-MDM2 and 14-3-3-MDMX, are shown to activate p53 upon stresses, and mutations of MDM2 or MDMX that may disrupt these pathways have been found in human cancers including HCC. Using our unique double knock-in (DKI) mice that contain wild-type p53 while harbor defects in these two pathways, we unveiled that basal level of p53 in DKI mice is sufficient for maintaining liver function, however, carcinogen- or unhealthy diet-induced HCC initiation is accelerated in DKI mice. The two p53-pathways are also activated in human cirrhotic livers. We futher identified a one-carbon metabolism (1CM) enzyme methylenetetrahydrofolate dehydrogenase 1 like (MTHFD1L) as the p53-suppressed player in HCC initiation. We found that MTHFD1L is upregulated in human early HCC and correlated with p53 status, and promotes mouse HCC initiation by enhancing autonomous growth and immune evasion of HCC initiating cells (HCICs). With Nanopore RNA-m^6^A-sequencing, we unveiled that MTHFD1L fulfills these bi-functions by fueling methionine cycle that produces methyl groups to maintain the mRNA m^6^A of Snail (an HCC early tumorigenesis driver) and B2m (a light chain of MHC-I required for antigen presentation to CD8+ T) in HCICs. Our findings demonstrate that the two stress-triggered p53-pathways are crucial for preventing liver early tumorigenesis by reversing MTHFD1L upregulation-associated 1CM reprogramming and subsequent Snail and B2m mRNA-m^6^A-mediated cell autonomous growth and immune evasion.

## INTRODUCTION

Hepatocellular carcinoma (HCC), the fifth most common cancer worldwide and the third leading cause of cancer-related deaths [1, 2], originates from injured hepatocytes that dedifferentiate into progenitor-like cells (or cancer initiating cells, CICs) expressing stem cell markers (e.g., CD44 and EpCAM). These cells expand to acquire the features of fully malignant cells in response to aberrant proliferative stimuli [3–6]. This transformation usually occurs in late-stage liver injury that involves closely intertwining stress responses and pathological processes, including endoplasmic reticulum (ER) and oxidative stresses, chronic inflammation, hepatocyte death, liver regeneration, somatic DNA alterations, fibrosis, and cirrhosis [7, 8].

In normal hepatocytes without stress, the tumor suppressor p53 level is kept low by MDM2 and MDMX that work together to inactivate p53 via a negative feedback [9]. However, during chronic liver injury, p53 activity is dynamically regulated. Early in regeneration, p53 is tightly controlled to enable the hepatocyte de-differentiation and division, but activated later to ensure the genomic integrity, keep the robustness and fidelity of cell proliferation, and prevent neoplasia transformation [4, 10, 11]. Several studies have shown that p53 inhibition by oncoproteins leads to early hepatocarcinogenesis [4, 12], suggesting that p53 must be activated prior to its inactivation. However, it remains unaddressed whether and how chornic liver injury-associated stress signals activate p53 under physiological conditions.

Two stress responsive signaling pathways can activate p53 by overcoming the MDM2/MDMX-mediated feedback: 1) the ribosomal proteins (RPs)-MDM2-p53 pathway, where ribosomal stress (RS) frees RPs such as RPL11 and RPL5 to bind the MDM2 central domain, and release p53 [13, 14]; 2) the 14-3-3-MDMX-p53 pathway, where DNA damage or glucose deprivation-induced MDMX phosphorylation enables 14-3-3 binding to block MDMX suppression of p53 [15, 16]. Both the pathways were independently validated by genetic knock-in (KI) mice: MDM2^C305F^ mice (M2-C305F) contain a point mutation at the central domain C305 position, to disable its binding to RPL5 and RPL11 [13, 17], and MDMX^3SA^ mice (MX-3SA) contain 3 serine mutations in the MDMX gene (Serine 367, 341 and 402) so that this MDMX mutant cannot be phosphorylated in response to stresses [15, 16]. Importantly, MDM2 truncations (AA1–302) of its central zinc finger domain (AA305–322) were previously identified in HCC [18], and our bioinformatic analysis also unveiled an MDM2 somatic point mutation (E316D) in this domain in HCC patients (Figure S24A). However, the role of these MDM2 central domain mutations in HCC has never been addressed. Besides, chronic liver injury-related stresses as mentioned above often induce ribosome dysfunction or activate kinases that phosphorylate MDMX at S342 or S367 [13–15, 19–22]. These studies highly suggest that the two stress responsive pathways may play a preventive role in HCC initiation.

To address this, we generated an MDMX/MDM2-double knock-in (DKI) mouse model by crossing the two aforementioned KI mouse lines, and induced HCC in the mice with administration of hepatic procarcinogen diethylnitrosamine (DEN). DEN-induced HCC mimics many of human HCC features [23]. The DKI mice exhibited significantly higher rates of DEN-induced HCC. HCC initiating cells (HCICs) isolated from these mice displayed enhanced tumorigenesis when introduced into recipient livers. DKI mice under a long-term high-fat diet (HFD) also developed more liver tumors. Moreover, the two p53-pathways were turned on in human livers with cirrhosis, the precancerous condition for HCC [24, 25]. We further identified methylenetetrahydrofolate dehydrogenase 1 like (MTHFD1L), a key mitochondrial one-carbon metabolism (1CM) enzyme producing formate [26], as a p53-suppressed player in HCC initiation. We found that Mthfd1l expression is declined in cirrhotic liver, while upregulated and highly correlated to the p53 mutation status in HCC patients. Mthfd1l depletion in DKI-HCICs significantly delayed HCC onset after intra-splenic transplantation. Furthermore, by fueling the methionine cycle MTHFD1L enhanced the N6-methyladenosine (m^6^A) modification of Snail [27, 28] mRNA to boost HCIC growth and stemness, as well as the modification of B2m (β2m) mRNA, a light chain of MHC-I molecules [29], which significantlly hampered CD8 + T liver tumor infiltration and cytoxicity against HCICs. Hence, our studies reveal a new MDM2/MDMX-p53-Mthfd1l-RNA m^6^A-Snail/B2m axis and its novel role in suppressing HCIC autonomous growth and immune evasion during HCC initiation, providing new insights into the regulation of p53-pathways on metabolic reprogramming, RNA epigenetics, and cancer immunoediting during cacer initiation. Our study also illustrates the bi-functions of MTHFD1L and its potential as a diagnostic biomarker and a therapeutic target for early HCC.

## RESULTS

### DEN induced-HCC occurs significantly higher in DKI than in CTL mice

To determine if the two p53-activating pathways play a role in preventing HCC initiation, we generated an M2-C305F/MX-3SA DKI mouse line (Figure S1). Control (CTL), DKI, M2-C305F and MX-3SA mice were obtained in a normal Mendelian ratio and employed for the experiments. All the mice were without significant defects at both postnatal and adult stages under normal conditions (Figure S1B).

**Figure 1:**
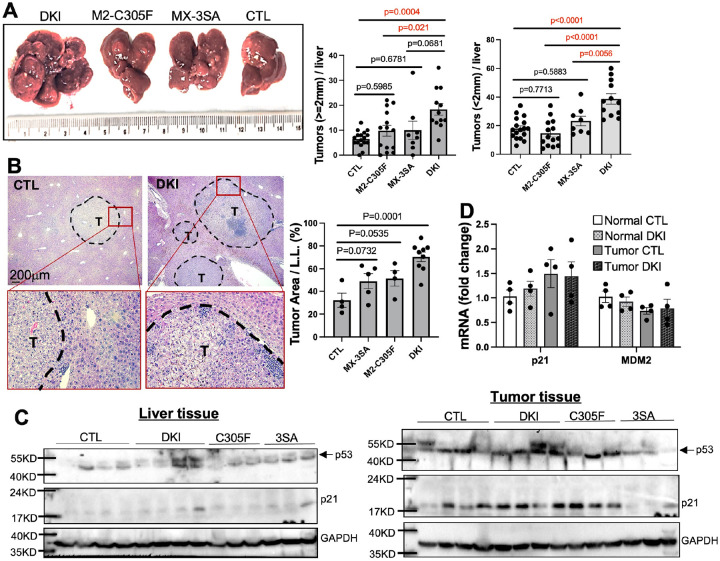
Male DKI mice show increased DEN-induced HCC incidence. Fifteen-day-old male mice were treated with DEN (25mg/kg), and livers were collected 9 months later. **(A)** Gross liver morphology and tumor nodule counts via macroscopy. **(B)** H&E staining on liver sections, and tumor area per large lobe (L.L.) quantified via ImageJ. **(C–D)** Western blot (C) and qPCR (D) analyses of tumor and adjacent tissues (n = 4 for qPCR). Data shown as mean ± SEM. (T, tumor)

We injected a single dose of DEN (25mg/kg) via i.p. into 2-week-old male and female mice [4]. All the males and most the females developed malignant multi-nodules in livers 9 months post-DEN (one female CTL had only one nodule). Consistent with previous reports [30, 31], the male mice developed significantly more and larger tumors than females (Figs. 1A and S2). In both sexes, DKI mice exhibited a significantly higher tumor number and/or larger average tumor area per large lobe than other groups (Figs. 1A–B and S2), indicating that DEN induced-HCC occurrence is enhanced in DKI mice with either sex. Surprisingly, the expression of p53 and its targets p21 and MDM2 at mRNA and protein levels in both tumor and adjacent liver tissues, was comparable among the groups (Figs. 1C–D). These results implicate that p53 might be activated through the two pathways at the early, but not advanced, stage of HCC to suppress DEN-induced HCC initiation.

### Pericentral hepatocyte proliferation and HCC initiation induced by DEN are significantly increased in DKI mice

**Figure 2:**
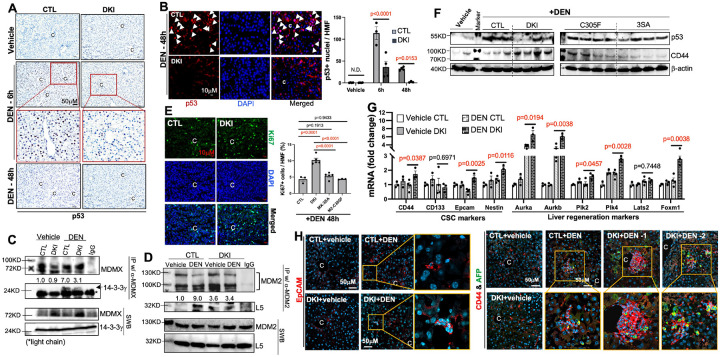
DKI mice show impaired p53 activation and accelerated HCC initiation after DEN treatment. **(A)** Liver sections from DEN (25mg/kg)-treated 15-day-old males were stained for p53. **(B)** 48h post-DEN, liver sections were stained with anti-p53, flowed by cyanine 3 (Cy3) TSA-plus incubation. p53+ nuclei in zone 3 per high magnification field (HMF) were quantified. (n=3–4/group) **(C–D)** Co-IP was performed on liver tissues 6h post-DEN. **(E)** Liver sections were stained for Ki67 to assess cell proliferation 48h post-DEN. (n=3–5/group) **(F–H)** Livers were harvested 1 (F-G) or 4 (H) months post-DEN for WB (F), qPCR (G), and immunostaining (H). (n=3–4/group) (C, central vein; ND, not detectable)

To test the speculation above, we examined if p53 is activated by DEN in 2-week-old male mouse livers. Indeed, p53 protein level and/or activity rapidly increased in CTL-livers 6 hours after DEN treatment and remained elevated at 48h, but not in DKI-livers (Figs. 2A–B and S3). Of note, p53 induction in CTL-livers was mainly localized to the pericentral hepatocytes (zone 3), aligning with reports showing DEN- or NASH-induced HCC originates from this zone [4, 5]. Furthermore, the binding of 14-3-3γ to MDMX or L5 to MDM2 was significantly enhanced in DEN-treated CTL-livers, but not in DKI-livers (Figs. 2C–D), indicating that both pathways contribute to the DEN-induced p53 activation. Also, Ki67 staining revealed significantly higher hepatocyte proliferation in zone 3 of DKI livers at 48h post-DEN (Fig. 2E), suggesting that p53 activation through these two pathways restrains the compensatory proliferation in CTL livers.

We then examined the liver tissues 1 and 4 months post-DEN, when damaged hepatocytes start to transform towards HCICs[3]. Remarkably, cancer stem cell (CSC) markers (CD44, EpCAM and Nestin [4, 11]) and liver regeneration markers were significantly elevated as early as 1 month post-DEN in DKI-livers, along with lower p53 level (Figs. 2F–G). Four months post-DEN, both CSC markers and malignancy markers (Ly6D and AFP) remained at a higher level in DKI-livers (Figs. 2H and S4), with more CD44 and AFP double-positive cells in zone 3 (Fig. 2H). Collectively, our *in vivo* findings demonstrate that p53 activation through these two pathways prevents DEN-induced HCC initiation in CTL mice.

### p53 suppresses the stemness and malignancy properties of DKI-HCICs

**Figure 3:**
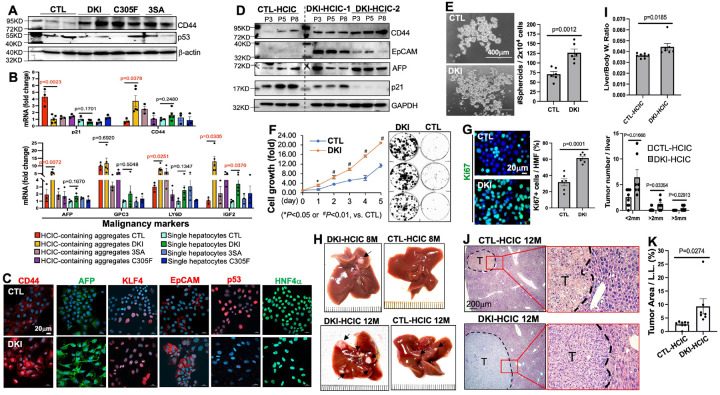
DKI-HCICs with reduced p53 show enhanced stemness, malignancy, and tumorigenicity. **(A–B)** HCIC-enriched clusters were isolated 3 months post-DEN and analyzed by WB (A) and qPCR (B, n=3–5/group). **(C–D)** Cultured HCICs were stained for cancer stem cell (CSC)/malignancy markers (C) and subjected to WB (D). **(E–G)** Cultured HCICs were assessed for spheroid formation (E), growth and colony formation (F), and proliferation (G). Experiments were repeated 4–5 times. **(H–K)** Freshly isolated CTL and DKI-HCICs were transplanted into C57BL/6J mice via intra-splenic injection. Liver morphology (H), liver/body weight ratio and nodule count (I), H&E staining (J), and tumor area (K, using *Image J*) were evaluated at 8 and/or 12 months post-transplantation. (n=7–8/group)

Next, we isolated HCIC-enriched cell clusters from male CTL and DKI-livers 3 months post-DEN as previously described [3, 32]. As expected, both fresh (Figs. 3A–B and S5A) and cultured (Figs. 3C–D and S5B) DKI-HCICs exhibited significantly higher levels of CSC markers (CD44, EpCAM, KLF4) and malignancy markers, but lower p53 and p21, compared to CTL-HCICs. All HCICs were HNF4α positive (Fig. 3C), confirming their hepatocyte origin. Furthermore, significantly more spheroids were formed with DKI-HCICs (Fig. 3E), and greater cell viability was obsereved in DKI-HCICs as detected by cell growth and colony formation assays (Fig. 3F), primarily attributed to the higher proliferation of DKI-HCICs (Fig. 3G). However, ectopic p53 markedly reduced their cell viability/growth and spheroid formation as well as the expression of CSC markers (EpCAM, CD44, KLF4 and SOX9) and malignancy marker AFP (Figure S6). These *in vitro* results demonstrate that p53 activation via the two pathways blocks the hepatocyte transformation towards HCICs.

### Elevated tumorigenic capacity of transplanted DKI-HCICs in vivo

We then transplanted fresh CTL- and DKI-HCICs into male C57BL/6 WT mice via intra-splenic injection (Figure S7) as previously described [3, 32]. All DKI-HCIC recipient mice (3 out of 3) developed a tumor nodule in each liver 8 months post-transplantation, and multiple tumor nodules 12 months later (7 out of 7) (Fig. 3H). Nevertheless, none of the CTL-HCIC recipients developed palpable liver tumors by 8 months after injection. Though 12 months later 7 out of 8 CTL-HCIC recipients developed liver tumors (Fig. 3H), these tumors were much less and significantly smaller than those in DKI-HCIC recipients (Figs. 3I and 3K). H&E staining further confirmed those nodules in both groups as HCC (Fig. 3J). Again, these results consolidate that impairment of the two p53-pathways significantly accelerates liver tumorigenesis.

### Mthfd1l is crucial for HCC initiation in DKI mice

**Figure 4:**
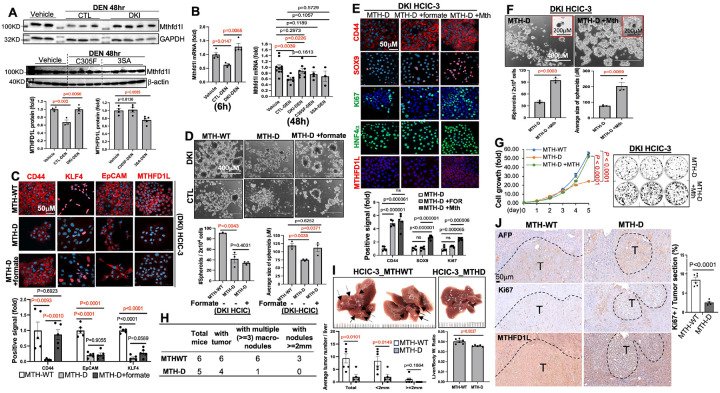
Mthfd1l is essential for accelerated HCC initiation in DKI mice. **(A–B)** Livers from DEN-treated 15-day-old males were analyzed for Mthfd1l expression by WB and qPCR at 6h or 48h. **(C–D)** CTL or DKI-HCICs with wild-type (MTH-WT) or CRISPR-depleted (MTH-D) Mthfd1l were treated with vehicle or formate (1 mM) for 48h and analyzed by immunostaining (C) and spheroid assay (D). Experiments were repeated 3–5 times for each HCICs. **(E–G)** MTH-D DKI-HCICs re-expressing Mthfd1l or vector control were subjected to IF staining (E), spheroid formation (F), and growth/colony formation assays (G). Experiments were repeated 3–5 times. **(H–J)** MTH-WT or MTH-D DKI-HCICs were transplanted into C57BL/6J mice via intra-splenic injection. After 10 months, liver morphology and tumor nodules were assessed (H–I), followed by IHC analysis (J).

To identify p53 downstream players in HCC initiation of DKI mice, we performed RNA-seq with DEN-induced liver tumors and adjacent liver tissues of CTL and DKI mice. PCA and heatmap analyses revealed that the overall gene expression pattern of DKI-livers is closer to that of CTL- or DKI- tumors (Figure S8A). Pathway enrichment highlighted significant alterations in metabolic pathways, especially the monocarboxylic acid metabolism that was among the top changed metabolic pathways in DKI-liver versus CTL-liver, or CTL-tumor versus CTL-liver (Figures S8B-C). Notably, Mthfd1l, an enzyme involved in 1CM to produce formate (a type of monocarboxylic acid), was significantly upregulated in DKI-livers, CTL-tumors, DKI-tumors, and all stages of human HCC (Figures S8D-E). Its higher levels were also associated with poor survival in patients with early-stage HCC (stage I) (Figure S8F). Mthfd1l expression in 15-day-old CTL-livers, but not in DKI-livers, were drastically reduced after DEN treatment (Figs. 4A and B). Four months post-DEN, DKI-livers presented a significantly higher formate levels (Figure S9). Furthermore, fresh or cultured DKI-HCICs showed significantly higher Mthfd1l expression or formate levels compared to CTL and/or SKI counterparts (Figure S10). These findings suggest that MTHFD1L-associated 1CM reprogramming might play a role in the accelerated HCC initiation in DKI mice.

To test this idea, we knocked out the Mthfd1l gene in CTL and DKI-HCICs using CRISPR/cas9 system. Mthfd1l was depleted in three cell lines (one CTL- and two DKI-HCIC lines from three individual mice) (Figure S11A). Nanopore direct RNA-seq showed a sharp drop of the read depth in MTHFD1L Exon 6 in HCIC-1 (CTL) and − 3 (DKI) cells, indicating Mthfd1l deficiency (MTH-D), however only 1 nucleotide deletion in MTH-D HCIC-2 (DKI) (Figure S11B), so we employed MTH-D HCIC-1 and − 3 cells for the following experiments. Mthfd1l deletion significantly diminished CSC marker expression and cell viability/growth, more so in DKI-HCICs (Figs. 4C, S11C-D and S12), and impaired spheroid formation in both the HCICs (Fig. 4D). We further tested whether formate supplementation could resuce these effects caused by Mthfd1l depletion. After formate treatment for 48h, CD44 expression in MTH-D HCICs was almost recovered to the level in Mthfd1l-proficient (MTH-WT) cells (Figs. 4C, 4E and S11C-D). The size, but not the number, of the spheroids was also rescued by formate (Fig. 4D). However, cell proliferation (Ki67 + signals) and growth showed partial or minimal recovery after formate treatment (Figs. 4E and S12B). These results implicate that while formate improves some Mthfd1l deletion-caused defects, Mthfd1l might also utilize a non-enzymatic mechanism to promote growth and self-renewal of HCICs. We re-expressed Mthfd1l in MTH-D DKI-HCICs to test if these defects can be rescued. Remarkably, replenishment of Mthfd1l restored CSC marker expression (CD44 and SOX9) (Figs. 4E), spheroid formation (Fig. 4F), cell viability (Fig. 4G), and proliferation (Ki67 signals in Fig. 4E) of MTH-D HCICs to near-normal levels. Furthermore, 10 months after intra-splenic injection, MTH-WT DKI-HCICs formed multiple tumors in recipient livers, while MTH-D cells produced significantly fewer palpable tumors, along with significantly lower liver/body weight ratio of MTH-D HCIC recipients (Figs. 4H–I). H&E and IHC staining confirmed that these tumors are AFP-positive HCC (Figs. 4J and S13) with significantly stronger Ki67 signals in MTH-WT DKI-HCIC-derived tumors (Fig. 4J). These results demonstrate that Mthfd1l activation owing to impairing the two p53 pathways in DKI mice is responsible for the accelerated HCC initiation.

### p53 transcriptionally suppresses Mthfd1l by cooperating with HDAC1/2

Our bioinformatic analysis showed that though Mthfd1l is upregulated in HCC patients, its expression is significantly lower in p53-wild type than in p53-mutant HCCs (Figure S14A). Indeed, Mthfd1l expression at mRNA and protein levels was p53-dependent, comparing p53-containing cells (HepG2 liver cancer and H460 lung cancer cells) with p53-null/-mutant cells (Figures S14B-F). p53 overexpression in DKI-HCICs reduced Mthfd1l expression (Figure S14G). These data indicate that p53 transcriptionally suppresses Mthfd1l.

Luciferase reporter assays using a reporter vector containing two putative p53 binding sites (BS) in the human MTHFD1L promoter (Mthfd1l-BS1/2) (Figure S15A left) showed that p53 activators reduces the luciferase signals by 50 ~ 60% only in HepG2 (Figures S15A right). This repression was abolished by BS1 mutation (Figure S15B), indicating that p53 inhibits MTHFD1L promoter activity via the Mthfd1l-BS1. Consistently, p53 binding to the MTHFD1L, p21, and Puma promoters was confirmed by ChIP assays in flag-p53-overexpressing Hep3B cells or in doxorubicin-treated HepG2 cells (Figure S15C).

P53 recruits an inhibitory complex with HDACs (Figure S16A) [33] to suppress its targets. Accordingly, treatment with the HDAC1/2 inhibitor TSA almost fully rescued Mthfd1l expression inhibited by the p53 activators only in HepG2, but not p53-null Hep3B, cells (Figure S16B). Consistently, the DNA-protein complexes pulled down by HDAC1/2 antibodies were enriched with the DNA fragments containing Mthfd1l-BS1 of p53 (Figure S16C). These results indicate that p53 represses Mthfd1l transcription by working with HDACs in HCC cells.

### MTHFD1L promotes HCIC autonomous growth and immune evasion via RNA mA methylation

**Figure 5:**
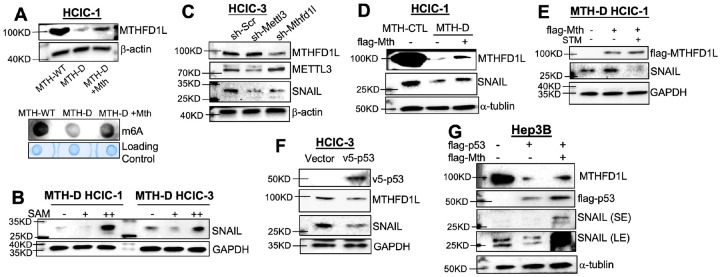
Mthfd1l promotes Snail mRNA m^6^A methylation to enhance HCIC proliferation. **(A)** MTH-WT and MTH-D HCICs were transfected with Mthfd1l or vector, followed by m^6^A Dot-blot. Experiments were repeated with 3 different HCIC cell lines, and representative images were shown. **(B-F)** WB was performed on MTH-WT or MTH-D HCICs with treatments or gene modifications including SAM (15 and 30 mM), Mettl3 or Mthfd1l knockdown, and Mthfd1l or p53 overexpression. **(G)** p53-null Hep3B cells were transiently transfected with Mthfd1l and/or p53, followed by WB.

Mthfd1l generates formate to supply ~ 75% of 1-C units in the methionine cycle [34] that produces direct methyl donor SAM for RNA m^6^A modificaiton. Indeed, in MTH-D HCICs, global mRNA m^6^A levels were drastically declined, but recovered by ectopic Mthfd1l (Fig. 5A). SAM significantly boosted CSC marker expression, spheroid formation, and growth of MTH-D HCICs (Figures S17A-C), while treatment with STM2457 (STM), an inhibitor of the major RNA m^6^A methylation writer methyltransferase like 3 (METTL3) [35], remarkably suppressed these traits of HCICs (Figures S17D-G). Also, our database analysis showed that a higher METTL3 level is well correlated with poorer survival in HCC patients (Figure S17H). These results suggest that MTHFD1L promotes HCC initiation by enhancing RNA m^6^A methylation.

**Figure 6:**
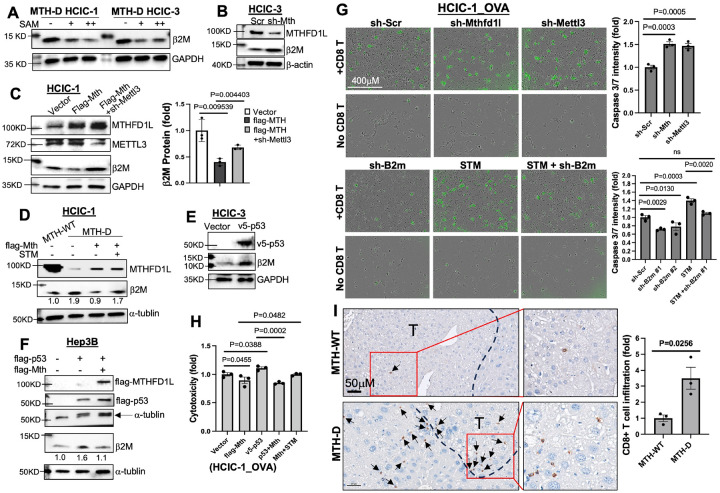
Mthfd1l promotes B2m mRNA m^6^A methylation in HCICs to suppress CD8+ T cell infiltration and cytotoxicity. **(A-E)** WB was performed on MTH-WT or MTH-D HCICs with treatments or gene modifications including SAM, Mettl3 or Mthfd1l knockdown, and Mthfd1l or p53 overexpression. Images in (A) were from the same WB of Figure 5B, and the samle loading control images were presented. Images in (E) were from the same WB of Figure 5F. **(F)** Hep3B cells were transiently transfected with Mthfd1l and/or p53, followed by WB. **(G)** After gene knockdown or STM pre-treatment, HCIC-1_OVA cells were co-cultured with OT-1 CD8+ Tcells, and caspase 3/7 activity (green fluorescence) was monitored under Incucyte. **(H)** HCIC-1_OVA cells transfected with Mthfd1l and/or p53 or pretreated with STM were co-cultured withOT-1 CD8+ T and tested by LDH cytotoxicity assay. **(I)** Sections of mouse liver tissue containing tumors derived from MTH-WT and WTH-D HCICtransplantation were stained with anti-CD8a antibody. Arrows indicate CD8+ T cells. (T, tumor area)

To test this idea, we performed Nanopore RNA m^6^A-seq with paired MTH-WT and MTH-D HCIC cells (HCIC-1 and HCIC-3). It showed a global reduction of RNA m^6^A in MTH-D cells (Figure S18A) and identified ~ 46 protein-coding genes with an average modification difference ≥ 5%. As one of the top 10 genes (Figures S18B-D), Socs2 was used as a positive control. Its decreased protein level due to the elevation of its mRNA m^6^A is linked to HCC progression [36]. Then the rest top candidates (Figure S18B) were screened by their response to SAM treatment in MTH-D HCICs (Figs. 5B and 6A). Snail, a well-known EMT player and newly identified HCC early tumorigenesis driver [27, 28], was upregulated by SAM (Fig. 5B). Conversely, in HCICs when knocking down Mthfd1l or Mettl3 (Fig. 5C), or in MTH-D HCICs when comparing with MTH-WT cells (Fig. 5D), Snail was dramatically reduced. These effects were reversed by re-expression of Mthfd1l in MTH-D HCICs (Figs. 5D–E), which was further withdrawn by STM treatment (Fig. 5E). P53 also suppressed Snail in both HCICs and Hep3B cells, but these effects were negated by Mthfd1l co-expression (Figs. 5F–G). However, knockdown of Mettl3 or Mthfd1l, re-expressing Mthfd1l in MTH-D HCICs, or treatment of STM exerted no effect on Snail mRNA level (Figures S20A-B). Besides, we found that the m^6^A mainly exists in the 3’-UTR region of Snail mRNA (Figure S18C). We further identified several m^6^A sites within that region. Those sites displayed m^6^A level > = 15% in MTH-WT HCICs and the modification decline > = 5% in MTH-D vs. MTH-WT cells (Figures S18C-D). Taken together, these findings demonstrate that the p53-MTHFD1L axis controls cell growth and stemness during HCC initiation, by modulating mRNA m^6^A to translationally upregulate Snail.

Hepatocytes express abundant cell surface MHC-I, and can rapidly load and present antigens[37, 38]. In MTH-D HCICs, the mRNA m^6^A level of β2-microglobulin (β2m, B2m), the light chain of MHC-I molecules, was reduced (Figures S18B-D). The nanopore m^6^A-seq analysis indicated a major m^6^A site in the coding region (CDS) of B2m mRNA in MTH-WT HCICs, which was decreased by 10% in MTH-D cells (Figures S18C-D). In contrast to the response of SNAIL to SAM treatment, the B2M protein level of MTH-D HCICs was dramatically decreased by SAM (Fig. 6A). Downregulation of B2m or MHC-I molecules dampens antigen presentation, impairs CD8 + T cell cytotoxicity and contributes to cancer immunotherapy resistance [29, 39]. Consistently, B2m is positively correlated with HCC patient survival (Figure S19). Also, Mthfd1l knockdown remarkably increased (Fig. 6B), while its overexpression suppressed, β2m expression in MTH-WT HCICs, which was rescued by Mettl3 knockdown (Fig. 6C). β2m protein level was doubled in MTH-D HCICs compared to MTH-WT cells, wheras Mthfd1l re-expression counteracted the higher β2m expression in MTH-D cells, which was recovered by STM treatment (Fig. 6D). P53 upregulated β2m in both HCIC and Hep3B cells (Figs. 6E–F), but this effect was eliminated by Mthfd1l overexpression (Fig. 6F). Notably, B2m mRNA levels were unchanged after Mthfd1l re-expression, STM treatment, Mthfd1l or Mettl3 knockdown in MTH-D or MTH-WT HCICs (Figures S20B-C), indicating a translational silence of m^6^A modified B2m mRNA. To validate the role of β2m in HCIC antigen presentation and subsequent CD8 + T cell activation, we co-cultured HCICs stably expressing chicken ovalbumin antigen (HCIC_OVA) (Figure S21A) with CD8 + T cells freshly isolated from C57 BL/6 OT-1 mouse spleen. OT-1 mice contain transgenic T cell receptor that recognizes OVA peptides presented by MHC-I. After co-culturing for 24h, CD8 + T cytotoxicity against HCIC, indicated by the caspase 3/7 signal, was enhanced by 1.5 times by Mthfd1l or Mettl3 knockdown in HCICs, while significantly weakened by B2m knockdown, which was mostly rescued by STM pre-treament on HCICs (Figures S21B and 6G). Furthermore, Mthfd1l overexpression in HCICs significantly inhibited p53-induced CD8 + T cytotoxicity, whereas STM pre-treatment reversed Mthfd1l-caused decline of CD8 + T cytotoxicity against HCICs (Figures S21B and 6H). Consistently, MTH-deficient (MTH-D) HCICs transplantation-derived liver tumors showed significantly increased CD8 + T cell infiltration *in vivo* (Fig. 6I). Together, these *in vitro* and *in vivo* results indicate that MTHFD1L promotes the immune evasion of HCICs by suppressing β2m-dependent antigen presentation and CD8 + T cytotoxicity via m^6^A-mediated translational control of B2m.

### Two p53-pathways play a preventative role in NASH- and cirrhosis-associated HCC initiation

**Figure 7:**
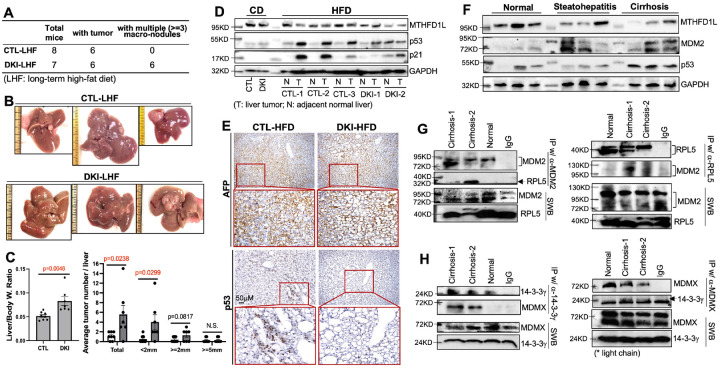
The two p53-activating pathways suppress HFD-induced HCC initiation and are activated in human cirrhotic livers. **(A–E)** CTL and DKI mice were fed a high-fat diet (HFD) for 15 months. Tumor incidence (A), liver morphology (B), liver/body weight ratio and tumor count (C), WB (D), and IHC (E) analyses were performed. (CD: chow diet) **(F–H)** Human liver samples (normal, steatohepatitis, and cirrhosis) were analyzed by WB (F) and Co-IP (G, H).

To determine if these two p53-pathways are triggered to prevent the NASH-associated HCC initiation, we fed CTL and DKI mice with a long-term HFD (LHF) for 15 months to mimic the human NASH-driven HCC. Both groups comparably developed severe steatosis and mild interstitial fibrosis (Figure S22), with tumors in ~ 75% of CTL and ~ 85% of DKI livers (Fig. 7A). Notably, all DKI mice developed multiple (≥3) tumor nodules, alongside with significantly higher liver/body weight ratio and more small nodules (diameter ≤ 2mm) in livers, whereas none of the CTL livers had more than two nodules (Figs. 7A–C). The HCC tumor size usually indicates tumor progression, while tumor number is an index of early tumorigenesis. Expectedly, the tumor nodules in both groups expressed a high level of AFP (Fig. 7E). However, p53 (and p21) was remarkably induced only in CTL-tumors compared to the adjacent liver tissues, but not in DKI-tumors, correlating with down-regulated MTHFD1L in CTL-tumors (Figs. 7D–E). Together, these data show that impairment of the two p53-pathways enhances NASH-driven HCC initiation.

We further assessed the p53-MTHFD1L signaling in human liver cirrhosis (Figure S23), the HCC precancerous condition [24, 25]. As shown in Fig. 7F, human alcoholic cirrhotic livers presented strong p53 induction, increased MDM2 and downregulated MTHFD1L. IP-WB analysis showed remarkably elevated formation of MDM2-RPL5 and MDMX-14-3-3γ complexes in cirrhotic versus normal livers (Figs. 7G–H). These results support an active role of the two p53-pathways in preventing human hepatocyte malignant transformation in cirrhosis by suppressing Mthfd1l.

## DISCUSSION

Using a DKI animal model, we have addressed the remaining crucial question: how is p53 rapidly activated prior to its inactivation during chronic injury to block HCIC transformation and HCC initiation? We for the first time identified a dual mode of p53 activation by inactivating both MDM2 and MDMX in response to various stresses to prevent HCC initiation. We showed that imparing the two p53-activating pathways markedly accelerates DEN- or NASH-driven HCC initiation in DKI mice (Figs. 1–2, S4, 7A–E and 8), DKI-HCICs exhibit higher malignancy and stemness (Figs. 3A–G) which can be reversed by p53 (Figure S6), and DKI-HCICs develop into HCC more severely (Figs. 3H–K). Correlatedly, p53 was activated through these two pathways in cirrhotic patient livers (Figs. 7F–H). About 80% of HCCs are developed from cirrhosis that is recognized as a precancerous state [24, 25]. In line with the literature, our results consolidate the biological significance of p53 activation through the two pathways in preventing HCC initiation.

While hepatocyte specific deletion of p53 spontaneously and consistently induces liver carcinoma formation [40], continuous p53 activation in hepatocytes (e.g., via MDM2 deletion) also promotes liver tumorigenesis [41], indicating that tight regulation on p53 activity is needed for preventing hepatocarcinogenesis. Different from other genetic models, our unique DKI mice retain WT p53, providing a special tool for studying p53’s function in real time. Indeed, p53 activation is observed mainly in the end-stage of liver disease, i.e. cirrhotic liver (Fig. 7F), or in early HCC stages, but blunted in DKI mice (Figs. 2F and 7D–E), suggesting a dynamic regulation of p53 activity by the two pathways during chronic liver injury and HCC initiation. Interestingly, similar to their SKI parents [16, 17], DKI mice are developmentally normal (Figure S1). Thus, the basal level of p53 in DKI mice is sufficient for maintaining normal liver function, but insufficient for protecting the animals from stresses-associated HCC tumorigenesis, highlighting the surveillance role of the two p53-pathways in early hepatocarcinogenesis long before somatic p53 mutations occur [4, 42, 43].

As aforementioned, mutations (Figure S24A) and truncations [18] of MDM2 central zinc finger domain that is critical for the RP binding were identified in HCC patients. In addition to HCC, other types of cancers also contain MDM2 somatic mutations in its central domain (Figure S24B). Over a dozen RPs and other proteins such as ARF, NPM, and Rb activate p53 under stresses by binding the MDM2 central domain, the same mode demonstrated in our current study, disrupting the MDM2-p53 feedback loop [14, 18, 44]. Similarly, somatic point mutations of MDMX at S367, S342 and other serine sites exist in human cancers (Figure S24C). Our study partially explains why the MDM2 central domain and the MDMX serine sites are mutated in various cancer patients including HCC, and reinforces the clinical significance of the dual-mode activation of p53 in preventing liver tumorigenesis.

One-carbon metabolism (1CM) is frequently reprogrammed in many cancers. Various enzymes for 1CM have been linked to different cancers including HCC, rendering the 1CM enzyme networks as very promising therapeutic targets of cancers [45]. Here, we identified the 1CM enzyme MTHFD1L as a new p53-suppressed downstream player in HCC initiation (Figs. 4, S8-S17, and 8). MTHFD1L could accelerate liver cancer progression by encountering oxidative stress [46]. Our bioinformatic analysis (Figure S8F) suggests that MTHFD1L plays a crucial role in HCC initiation. Consistently, Mthfd1l deletion drastically retarded HCIC tumorigenesis *in vivo* (Fig. 4). Interestingly, Mthfd1l is downregulated and well correlated with increased p53 in human cirrhotic livers (Fig. 7F). Given Mthfd1l is usually overexpressed in all stages of HCC (Figure S8E), Mthfd1l might serve as a diagnostic marker and a therapeutic target for early HCC.

Besides enhanced autonomous growth mechanisms[4], initiaiting-stage cancers gradually acquire an immune evasion ability to progress to advanced stages [39]. We demonstrate that impairing the p53-pathways leads to higher expression of Mthfd1l that utilizes both the mechanisms to accelerate HCC initiation (Figures S14–16, 4–6 and 8). By producing mitochondrial formate, MTHFD1L fuels the methionine cycle to maintain the high level of RNA m^6^A (Figs. 5A and S18A) that is observed in human HCC[36] and critical for HCIC stemness and proliferation (Figure S17). Specifically, MTHFD1L supports the m^6^A modification of Snail mRNA (Figures S18C-D), resulting in translational upregulation of Snail (Figs. 5 and S20A-B). Studies have established that Snail plays an essential role in HCC onset by inhibiting hepatocyte-differentiation transcriptional factors and promoting cell proliferation [27], and disrupting bile acid homeostasis [28]. Interestingly, overexpression of Snail in hepatocytes can independently lead to steatohepatitis and HCC onset [28]. As aforementioned, hepatocytes can express abundant cell surface MHC-I molecules for antigen presentation to CD8 + T cells[37, 38]. In a study, loss of heterozygosity of HLA alleles is identified in 17% of multifocal HCC cases to dampen the neoantigen presentation and enhance the tumor cell immune escape[47]. Here, we found that B2m is translationally repressed by the MTHFD1L-mediated mRNA m^6^A modification in HCICs (Figs. 6 and S20B-C). In line with the critical role of B2m in MHC-I-mediated antigen presentation to CD8 + T cells and subsequent T cell recognition [29], MTHFD1L restrained the CD8 + T liver tumor infilatration and cytotoxicity against HCICs (Figs. 6G–I), leading to reinforced immune evasion of HCICs, while p53 inhibition of Mthfd1l prevented this cancer immunoediting during HCC initiation (Figs. 6E, F, and H), providing new insights into the p53’s function of reversing cancer metabolic reprogramming to block cancer immunoediting.

RNA m^6^A modification is a reversible process regulated by writers (such as METTL3/14, WTAP, etc.), erasers (such as FTO and ALKBH5), and readers (such as IGF2BPs, YTHDFs, etc.), affecting RNA splicing, nuclear export, degradation, stability, and translation [48]. The fate of m^6^A modified mRNAs largely depends on the specific readers and modification regions. For instance, YTHDF2 promotes mRNA degradation, IGF2BPs enhances mRNA stability or translation [48], and m^6^A in the mRNA coding region can delay translation elongation [49]. We found the differential regulation of MTHFD1L on the m^6^A-mediated Snail and B2m expression (Figs. 5B–G, 6A–F, S20, and 8). It remains to identify the specific m^6^A readers and erasers modulating these targets during HCC initiation.

**Figure 8:**
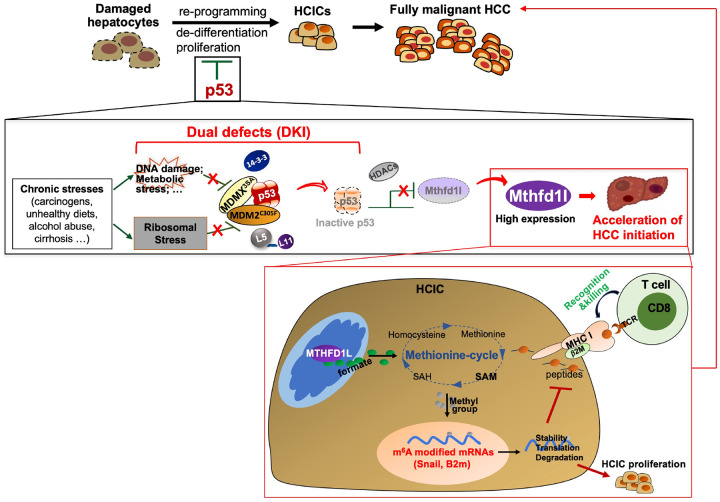
Model of the Stress - RPs-MDM2 / 14–3-3-MDMX -p53-Mthfd1l-RNA m^6^A-Snail/B2m axis in HCC initiation. In DKI mice, impaired p53 response to stress from carcinogens, NASH, or cirrhosis leads to reduced suppression of Mthfd1l. Elevated Mthfd1l promotes HCIC proliferation and immune evasion, accelerating transformation into HCC. (Red “X”s indicate disrupted pathway; arrows present activation; bars present inhibition.)

In summary, our study for the first time unveils the surveillance role of the two p53-pathways in averting MTHFD1L-accelerated HCC initiation upon pathologival signals, e.g., carcinogen, NASH, or alcoholic cirrhosis, and the novel bi-functions of MTHFD1L in promoting HCIC proliferation/stemness while suppressing immune elimination during HCC initiation, by fueling the methionine cycle to support RNA m^6^A modifications (Fig. 8). It also positions MTHFD1L and its downstream m^6^A targets as promising candidates for early diagnosis, prevention or therapy of HCC.

## METHODS

### Sex as biological variants

Both male and female mice were recruited for the DEN-induced HCC model, and similar findings were reported for both sexes, with significantly higher HCC incidence in the male mice, which is consistent with other animal studies and human HCC epidemiologic studies [30, 31, 50]. Male mice were used in other animal experiments due to their higher sensitivity and stability to HCC induction, and liver samples from female patients were purchased, as detailed below and in the *supplemental information*.

### Animals

#### Mouse lines.

M2-C305F and MX-3SA KI mouse lines were generously provided by Jeoff Wahl at SALK institute and Yanping Zhang at UNC at Chapel Hill, respectively [16, 17]. M2-C305F/MX-3SA-DKI mouse line was generated by crossing the two single-KI (SKI) mice as detailed in the supplemental information. Both male and female animals were used in this study and maintained in a C57BL/6 genetic background.

#### DEN-induced HCC mouse model.

To induce HCC, 15-day-old males and females were injected intraperitoneally (i.p.) with a single dose of DEN (25 mg/kg) and livers were collected 9 months later, following previous studies [4]. For some experiments involved in acute DEN treatment, 15-day-old male mice were i.p. injected with a single dose of DEN (25 mg/kg), and livers were collected at indicated time points for further analysis.

#### High-fat diet (HFD)-induced non-alcoholic steatohepatitis (NASH) and HCC mouse model.

Male control (CTL) and DKI mice were weaned at 3 weeks and fed with HFD (with 60 kcal% fat) (Research Diets, Inc.) and water *ad libitum* for 15 months.

#### Intra-splenic transplantation of HCICs.

C57BL/6 wild-type (WT) mice recruited for HCIC transplantation were from the Jackson Laboratory (Stock# 000664). Briefly, by following previous studies [3, 32, 51], 4-week-old male C57BL/6J WT mice were i.p. injected with retrosine twice with a 2-week interval. 4 weeks after the 2nd- retrosine treatment, each mouse received 1.5⋅10^5^ freshly isolated viable cells that were enriched with CTL- or DKI-HCICs, or 1·10^6^ cultured DKI-HCICs (passage 6, P6) with or without Mthfd1l gene deletion depending on experiments, in 100μl PBS via intra-splenic injection. One week after transplantation, CCl4 was given to the recipient mice intraperitoneally once a week for 3 weeks. Liver tissues of the recipient mice were harvested 8 ~ 12 months later for further analysis. All mice were housed under specific pathogen-free conditions with normal diet and water *ad libitum*.

### OT-1 mice and CD8 + T cell isolation

Two-month-old male C57BL/6 OT-1 mice were purchased from the Jackson Laboratory (Stock# 003831). Briefly, following previous study [52], OT-1 mouse spleen was homogenized and cell suspension was filtered through a 70μm cell strainer (Fisher), and erythrocytes were lysed. Then the single-cell suspension was centrifuged and counted, and untouched CD8 + T cells were purified using a mouse CD8a + T Cell Isolation Kit according to the manufacturer’s instructions.

### Primary cell isolation and culture

Primary HCICs and hepatocytes were isolated from male CTL and DKI mice treated with DEN or Vehicle, by following previous studies [3, 32, 51]. To ensure that enough HCICs can be obtained from mice for experiments and transplantation, male mice were used for DEN treatment. Fifteen-day-old mice were injected with one dose of DEN (25 mg/kg), and 3 months later mouse livers were perfused via inferior vena cava with warmed S&M buffer (0.24% Hepes, 0.019% NaOH, 0.05% KCl, 0.83% NaCl, pH7.4) and digest solution for 5min each, and minced in cold PBS. The cell aggregates containing HCICs were then isolated by sequential filtration with 70μm and 40μm cell-strainers, and subjected to gentle pipetting in cold PBS for intra-splenic injection.

For some experiments, HCICs were maintained in collagen-coated Petri dishes in DMEM medium (Gibco) supplemented with 20% fetal bovine serum (FBS) (Sigma), 1· PSN antibiotic mixture (10 ml/L) (Gibco), 20μg/L EGF, 0.01g/L Insulin, 1μm Dexamethasone and 1% L-glutamine. HCICs of passage 3–8 (P3-P8) were used for experiments to ensure the cell stemness.

### CRISPR/Cas9 mediated gene modification

HCIC cells with stable depletion/deletion of Mthfd1l (MTH-D) were generated using lentiviral CRISPR-Cas9 system. In short, three single guide RNAs (sgRNAs) (*Table S2)* respectively targeting exon 1, 6 and 12 of Mthfd1l gene were designed using online software (IDT), and inserted in plasmid LentiCRISPRv2 expressing wild-type Cas9, then co-transfected into HEK 293T cells with packaging plasmids following the *ZhangLab* protocol (https://media.addgene.org/cms/files/Zhang_lab_LentiCRISPR_library_protocol.pdf). CTL or DKI-HCICs (P3) were infected with lentivirus, and puromycin was added 48h later for selection of HCIC cells stably expressing Mthfd1l sgRNAs and Cas9. Single cell colonies were selected and cultured, followed by WB analysis and RNA sequencing to confirm the deletion. HCIC single colonies expressing Cas9 and WT-Mthfd1l (MTH-WT) were also simultaneously screened after infected with empty LentiCRISPRv2, to serve as paired control cells.

To establish MTH-D HCIC cell lines re-expressing Mthfd1l or HCICs expressing chicken ovalbumin (cOVA) (HCIC_OVA), the cells were infected with lentivirus expressing GFP-flag-Mthfd1l or cOVA, respectively. G418 or puromycin was added 48h later for Mthfd1l selection for 10 days or cOVA selection for 5 days, respectively.

### In vitro CD8 + T cytotoxicity assay

Briefly, ~ 4,000 HCIC_OVA cells/well were seeded in triplicate into a 96-well flat-bottom plate. The next day, freshly isolated C57BL/6 OT-1 CD8 + T cells were added to the wells (T cell: HCIC ratio is about 5:1) and co-cultured in complete DMEM growth medium for 24h in an Incucyte (Sartorius) culture chamber. NucView 488 Caspase 3/7 substrate (Biotium) was simultaneously added to each well, and caspase 3/7 positive signal (indicating the amount of T-cell killing) were measured following previous studies[53]. Five images per well were captured every hour using a 10× objective lens. Caspase 3/7 fluorescence intensity was quantified using the Incucyte built-in software combined with *Image J*. In those Control wells, HCIC_OVA cells were cultured without CD8 + T.

In some experiments, HCIC_OVA cells were co-cultured with OT-1 CD8 + T for 24h, followed by the measurement of released lactate dehydrogenase (LDH) with a LDH Cytotoxicity Assay kit (ThermoFisher) according to the manufacturer’s instructions.

### Statistics

All in vitro experiments were performed in at least triplicate. Data were presented as means ± SEM with N being the sample size. Comparisons among different groups were conducted by using One-way ANOVA or Student’s two-tailed t-test. Probability values of *P* ≤ 0.05 were considered statistically significant.

Supplementary information is available at *Cell Death and Differentiation*’s website.

## Supplementary Material

Supplementary Files

This is a list of supplementary files associated with this preprint. Click to download.

- RawimagesforWBDKIHCC02222026.pdf
- supplinfoforCDD.pdf

## Data Availability

GEO accession number for Nanopore RNA m^6^A-seq data is GSE310323. GEO accession number for RNA-seq data will be available upon submission. All raw data of immunoblotting images generated in this study will be deposit and made publicly available upon publication of this manuscript. Any additional information required to reanalyze the data reported in this paper is available from both the corresponding authors upon reasonable request.
